# A Mobile App for Stress Management in Middle-Aged Men and Women (Calm): Feasibility Randomized Controlled Trial

**DOI:** 10.2196/30294

**Published:** 2022-05-24

**Authors:** Breanne Laird, Megan Puzia, Linda Larkey, Diane Ehlers, Jennifer Huberty

**Affiliations:** 1 Arizona State University Phoenix, AZ United States; 2 Behavioral Research and Analytics, LLC Salt Lake City, UT United States; 3 University of Nebraska Medical Center Omaha, NE United States

**Keywords:** stress, meditation, mHealth, COVID-19, mobile app, mental health, mindfulness, digital intervention, psychological outcomes

## Abstract

**Background:**

Middle-aged adults (40-65 years) report higher stress levels than most other age groups. There is a need to determine the feasibility of using a meditation app to reduce stress and improve stress-related outcomes in middle-aged adults with a focus on men, as previous meditation app–based studies have reported a low proportion of or even no male participants.

**Objective:**

This study aims to (1) determine the feasibility (ie, acceptability and demand with a focus on men) of a consumer-based meditation app (Calm), to reduce stress among middle-aged adults reporting elevated stress levels, and (2) explore the preliminary effects of Calm on perceived stress, psychological outcomes (anxiety, depressive symptoms, mindfulness, and general coping), health behaviors (physical activity and eating habits), and COVID-19 perceptions.

**Methods:**

This feasibility randomized controlled trial evaluated an app-based meditation intervention in middle-aged adults (N=83) with elevated stress levels (ie, Perceived Stress Scale score ≥15) and limited or no previous experience with meditation. Participants were randomized to the intervention group (Calm app) or a control (educational podcasts; POD) group. Participants completed self-report assessments at baseline and postintervention (week 4). Feasibility was measured as acceptability and demand using Bowen framework. Feasibility and COVID-19 perceptions data were examined using descriptive statistics, and preliminary effects were evaluated using repeated measures analysis of variance.

**Results:**

Participants were satisfied with Calm (27/28, 96%) and found it appropriate or useful (26/28, 93%). Most reported they would likely continue using the Calm app (18/28, 64%). More Calm users reported satisfaction, appropriateness or usefulness, and intent to continue app use than POD users. Calm users (n=33) completed a mean of 20 (SD 31.1) minutes of meditation on the days they meditated and 103 (SD 109.1) minutes of meditation per week. The average adherence rate to the prescribed meditation was 71% among Calm app users, compared to 62% among POD users. Recruitment rate of men was 35% (29/83). Of those randomized to Calm, 55% (15/29) were men, and retention among them was higher (14/15, 93%) than that among women (12/20, 60%). No significant within or between group differences were observed.

**Conclusions:**

A 4-week, app-based mindfulness meditation intervention (Calm) may be feasible for middle-aged adults and a useful stress-management tool. Calm users expressed satisfaction with the app and felt it was appropriate and useful. Significant improvements in perceived stress and psychological outcomes or stress-related health behaviors were not observed. Even though men spent less time in meditation than women did and completed fewer weekly sessions, they were more likely to adhere to the prescription. Further research is needed for improving stress and stress-related outcomes among middle-aged adults with emphasis on the effects of mindfulness meditation apps for men.

**Trial Registration:**

ClinicalTrials.gov NCT04272138; https://clinicaltrials.gov/ct2/show/NCT04272138

## Introduction

Middle-aged adults (40-65 years) [1] report higher stress levels than most other age groups [2,3], with 75% reporting moderate to high stress and 33% reporting extreme stress [4]. Major sources of stress include managing children, employment, and aging parents [5]. In 2020, 78% of middle-aged adults reported the COVID-19 pandemic as a significant source of stress, and 67% reported increased stress during the pandemic [6]. When left unmanaged, stress is a risk factor for age-related chronic health conditions [7].

Meditation is the most prevalent nonpharmacological approach known to reduce stress [8,9]. Meditation interventions of only 5 to 10 minutes for 3 to 4 sessions a week can buffer reactivity to stress [10]. The use of smartphone apps to deliver meditation and manage stress is rapidly increasing [11] owing to their reach, accessibility, and low cost [12-15]. Meditation apps may overcome in-person participation barriers, such as travel, time, costs, stigma, and risk of infectious disease [16-18].

Engagement in health behaviors (eg, physical activity and eating habits) and treatment preferences for stress reduction often differ between men and women [19]. Previous studies on meditation apps have reported a low percentage of men in their samples [20,21], whereas some studies did not include men at all [22-24]. Feasibility and efficacy studies using the meditation app Calm have been conducted [25,26], but this is the first study testing Calm specifically in middle-aged adults with a focus on recruitment of men. There is a need to conduct additional feasibility studies using meditation apps to reduce stress and improve stress-related outcomes in middle-aged men and women [27].

The aims of this study were to (1) determine the feasibility (ie, acceptability and demand with a focus on men) of a consumer-based meditation app (ie, Calm), to reduce stress in middle-aged adults reporting elevated stress levels, and (2) explore the preliminary effects of Calm on perceived stress, psychological outcomes (ie, anxiety, depressive symptoms, mindfulness, and general coping), health behaviors (ie, physical activity and eating habits), and perceptions of COVID-19.

## Methods

### Research Design and Participants

This was a randomized controlled feasibility study approved by an institutional review board (STUDY00011219; NCT04272138). Middle-aged adults with elevated stress levels (Textbox 1) were recruited for an “app-based health and well-being study,” via flyers encouraging men to participate shared on social media platforms (eg, Instagram and Facebook) and the *ResearchMatch* website [28]. All participants provided electronic consent. Eligibility, consent, demographic, and survey data were collected using REDCap (Research Electronic Data Capture), a secure, web-based software platform, hosted by Arizona State University [29,30].

Participants were randomized to an app-based meditation intervention (Calm) or an app-based education control group (POD, an app that delivered podcasts on health and well-being, but excluding mindfulness, stress, or sleep content in the same context that a consumer-based mindfulness meditation app delivers content [31]) by using a randomized numbered list generated through simple randomization via the *Research Randomizer* website [32]. Participants were asked to complete 10-minute meditations daily on the Calm app or to listen to 10-minute educational podcasts daily on the POD app, for 4 weeks. All participants received weekly reminders and links to REDCap assessments via email.

**Eligibility criteria for participation in the study.**

**Inclusion criteria**

- man or woman
- age 40-64 years
- report a score of ≥15 on the Perceived Stress Scale
- have access to a smartphone on a daily basis
- willing to download the Calm app
- willing to be randomized to a meditation group or a health education podcast control group

**Exclusion criteria**

- have practiced mindfulness for >60 minutes/month in the last 6 months
- currently using the Calm app or another meditation app
- currently prescribed mood medication(s)
- currently residing outside the United States

### Measures

Participants completed self-report assessments at baseline and postintervention (ie, week 4). Feasibility was measured as acceptability and demand using the Bowen framework (Table 1) [27], and feasibility benchmarks were established using a previously established methodology of feasibility trials with Calm [25,33]. Benchmarks for acceptability were more than 75% of participants reporting each of the following: satisfaction with the intervention, perceiving the app as appropriate and useful, and intent to continue using the app. Benchmarks for demand were more than 40% of the sample comprising male participants, more than 70% of the Calm app user group adhering to at least 70% of the meditation intervention (ie, ≥10 minutes/day of using Calm), and more than 75% retention of men allocated to the intervention group. Adherence was measured using objective app usage data collected by both Calm and POD. The measures used to explore preliminary effects and COVID-19 perceptions are listed in Table 2. The COVID-19 Perceptions Survey was added to the postsurveys in March 2020 following the start of recruitment.

**Table 1:** Feasibility outcome measures.

Outcome	Measure	Acceptability	Demand	Baseline	Postintervention
Satisfaction	Satisfaction survey	✓			✓
Appropriate and useful	Satisfaction survey	✓			✓
Intent to continue use	Satisfaction survey	✓			✓
Recruitment of men	Demographics survey		✓	✓	✓
Adherence	App usage data		✓	✓	✓
Retention of men	Postintervention surveys		✓		✓

**Table 2:** Self-reported outcome measures.

Outcome	Measure	Baseline	Postintervention
Demographics	Demographics survey	✓	
Perceived stress	Perceived Stress Scale [34]	✓	✓
Anxiety	Hospital Anxiety and Depression Scale [35]	✓	✓
Depression	Hospital Anxiety and Depression Scale [35]		
Mindfulness	Mindful Attention Awareness Scale [36]	✓	✓
Physical activity	International Physical Activity Questionnaire Short Form [37]	✓	✓
	
Eating habits	Salzburg Stress Eating Scale [38]	✓	✓
General coping	Brief COPE^a^ [39]	✓	✓
COVID-19 perceptions	COVID-19 Perceptions Survey		✓

^a^COPE: Coping Orientation to Problems Experienced (Scale).

### Statistical Analysis

Feasibility and COVID-19 perceptions data were examined using descriptive statistics. Preliminary effects on perceived stress, anxiety, depressive symptoms, mindfulness, coping, physical activity, and eating habits were examined using repeated measures analysis of variance. Data were analyzed in SPSS (version 26; IBM Corporation) and SAS (version 9.4; SAS Institute). Results were considered significant at a 2-tailed *α* value <.05.

## Results

### Overview

**Figure 1:**
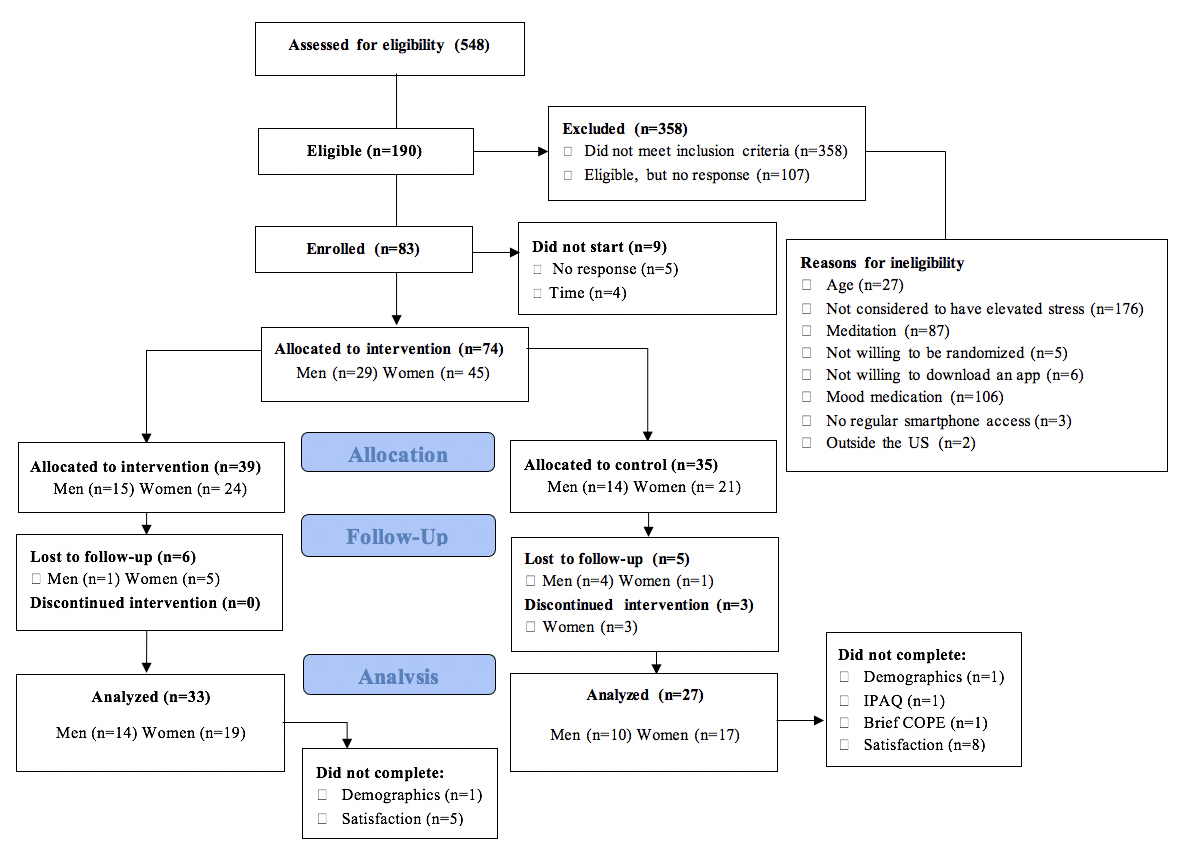
Enrollment of participants in the study. Note: Some participants did not meet more than one inclusion criteria. COPE: Coping Orientation to Problems Experienced (Scale); IPAQ: International Physical Activity Questionnaire.

In total, 83 middle-aged adults were consented and randomized, of which 60 (72%) were included in the analysis (Figure 1). Demographic characteristics of the sample are presented in Table 3.

**Table 3:** Baseline demographics of study participants.

Characteristic		Calm group (n=32)^a^	POD group (n=26)^a^	*P* value
			
Age in years, mean (SD)		52.1 (6.7)	50.8 (6.9)	.18
**Gender, n (%)**				.59
	Male	12 (37.5)	8 (30.8)	
	Female	20 (62.5)	18 (69.2)	
**Education, n (%)**				.67
	Bachelor's degree or higher	22 (68.7)	20 (76.9)	
**Ethnicity, n (%)**				.41
	Hispanic	3 (9.4)	1 (3.8)	
	Non-Hispanic	29 (90.6)	25 (96.2)	
**Race, n (%)**				.27
	White or Caucasian	27 (84.4)	19 (73.1)	
	Asian or Asian American	1 (3.1)	0 (0)	
	Black or African American	4 (12.5)	7 (26.9)	
**Income, n (%)**				.57
	US $61,000 or higher	22 (68.8)	16 (61.5)	
	US $60,000 or lower	10 (31.2)	10 (38.5)	
**Marital status, n (%)**				.41
	Married	23 (71.9)	13 (50)	
	Single	5 (15.6)	6 (23.1)	
	Divorced	2 (6.3)	2 (7.7)	
	Partnered	2 (6.3)	4 (15.4)	
	Separated	0 (0)	1 (3.8)	
**History of PTSD^b^, n (%)**				.53
	Yes	7 (21.9)	4 (15.4)	
	No	25 (78.1)	22 (84.6)	
**History of depression, n (%)**				.60
	Yes	8 (25)	5 (19.2)	
	No	24 (75)	21 (80.8)	
**Health status, n (%)**				.11
	Excellent	3 (9.4)	2 (7.7)	
	Very good	6 (18.8)	11 (42.3)	
	Good	14 (43.8)	10 (38.5)	
	Fair	8 (25)	1 (3.8)	
	Poor	1 (3.1)	2 (7.7)	

^a^One participant did not complete the survey.

^b^PTSD: posttraumatic stress disorder.

### Feasibility

#### Acceptability of Calm

Participants were satisfied with the meditation intervention (27/28, 96%) and found it appropriate or useful (26/28, 93%). Most participants reported that they were likely to continue using Calm in the future (18/28, 64%; Table 4). There were no notable differences in satisfaction, appropriateness or usefulness, or intent to continue use by gender (Table 5). More participants in the Calm group reported satisfaction, appropriateness or usefulness, and intent to continue use than in the control group (Table 4).

**Table 4:** Acceptability results of the Calm app classified by study group.

Question	Value, n (%)	
	Calm group (n=28)^a^	POD group (n=19)^b^
Overall satisfaction with study	27 (96.4)	10 (52.6)
Participation of the app was appropriate and useful	26 (92.9)	8 (42.1)
Would continue to use the app	18 (64.3)	6 (31.6)
Reduced stress in short term	16 (57.1)	7 (36.8)
Will help reduce stress in long term	19 (67.9)	10 (52.6)
Increased awareness of the importance of addressing stress	23 (82.1)	12 (63.2)
Will help reduce stress in the future	17 (60.7)	4 (21.1)
Likely to recommend the app to others	22 (78.5)	8 (42.1)

^a^Five participants did not complete the survey.

^b^Eight participants did not complete the survey.

**Table 5:** Acceptability results of the Calm app classified by participants’ gender.

Question	Value, n (%)		
	Female (n=19)^a^	Male (n=9)^b^	
Overall satisfaction with study	18 (94.7)	9 (100)	
Participation of the app was appropriate and useful	18 (94.7)	8 (88.9)	
Would continue to use the app	18 (64.3)	14 (73.7)	
Reduced stress in the short term	16 (57.1)	10 (52.6)	
Will help reduce stress in the long term	15 (79)	4 (44.4)	
Increased awareness of the importance of addressing stress	15 (78.9)	8 (88.9)	
Will help reduce stress in the future	19 (100)	8 (88.9)	
Likely to recommend the app to others	22(78.5)	16(84.2)	

^a^Five participants did not complete the survey.

^b^Eight participants did not complete the survey.

#### Demand of Calm (Adherence, Recruitment of Men, and Retention of Men)

**Figure 2:**
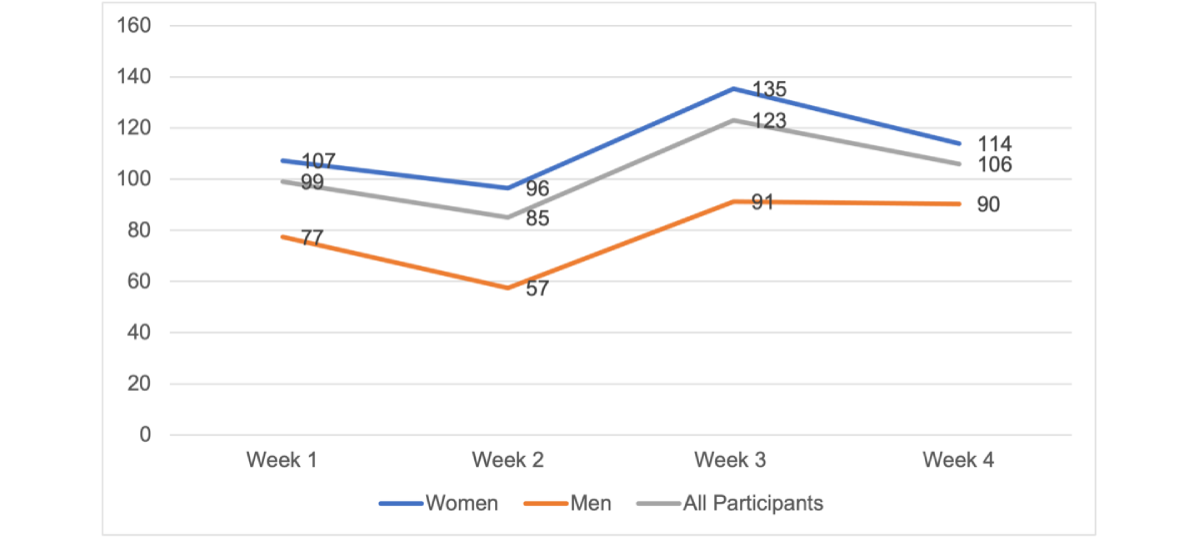
Average meditation time (in minutes) per week.

**Figure 3:**
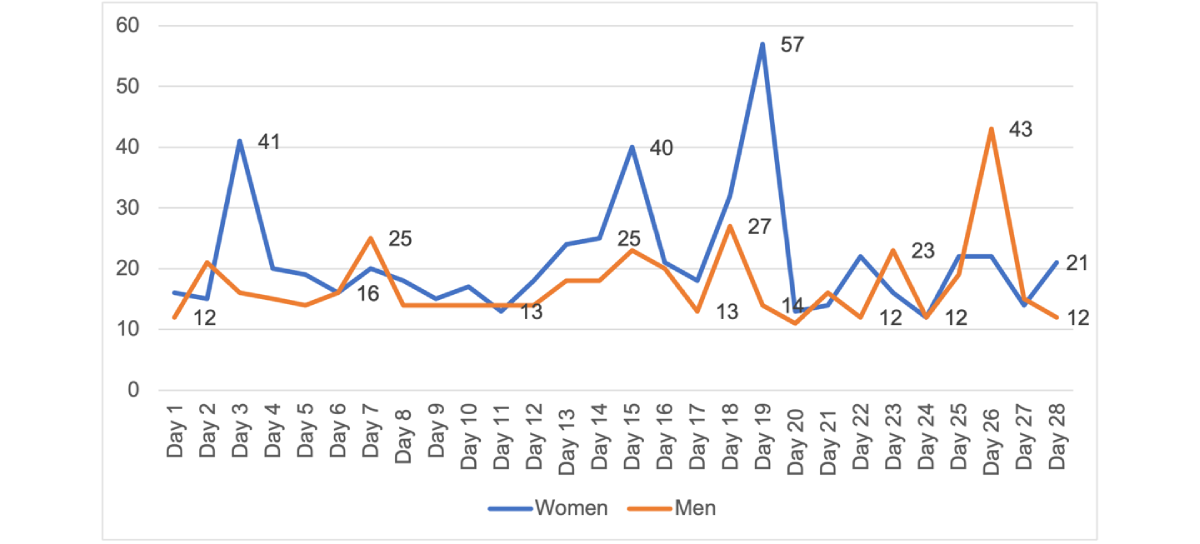
Average daily meditation time (in minutes) by gender.

**Figure 4:**
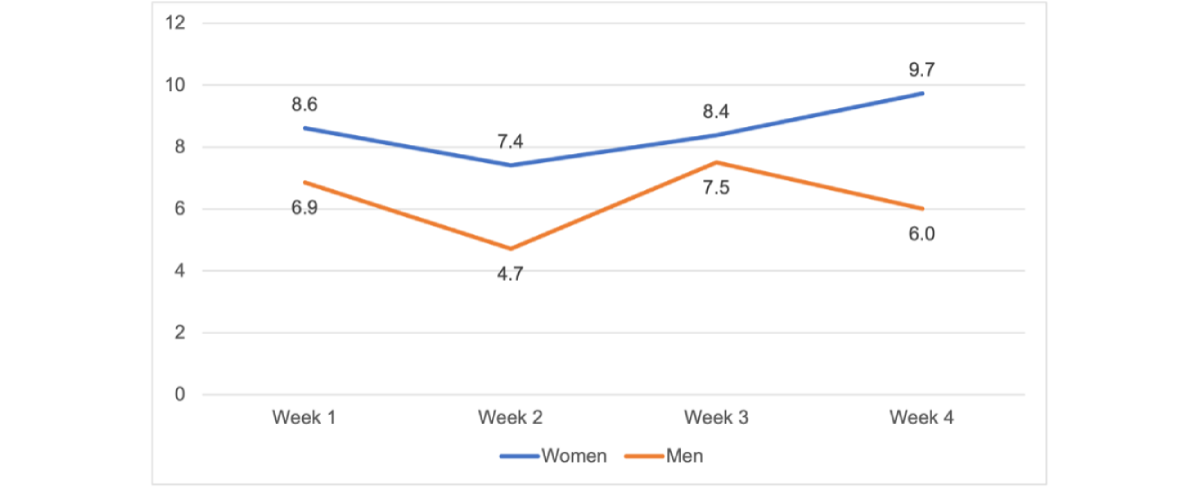
Average weekly meditation sessions by gender.

Calm participants (n=33) completed a mean of 20 (SD 31.1) minutes of meditation on the days they meditated, and a mean of 103 (SD 109.1) minutes of meditation per week during the study (Figure 1). On average, the adherence rate was 71% in the Calm group (ie, those who completed at least 70% of the meditation prescription) to the prescribed meditation, compared to 62% in the POD group. Men (n=14) completed a mean of 17.3 (SD 14.6) minutes of meditation per day (on the days they meditated), 79 (SD 37.9) minutes of meditation per week, and 8.5 (SD 5.7) meditation sessions per week during the study. Women (n=19) completed a mean of 21.5 (SD 31.1) minutes of meditation per day, 113 (SD 126.6) minutes of meditation per week, and 6.3 (SD 2.7) meditation sessions per week. Men showed greater adherence (12/14, 86%) to the Calm app than did women (12/19, 63%; Figures 2-4).

Recruitment rate of men into the study (before excluding those who were randomized and had completed baseline but did not start the study, those who dropped out, or those who did not complete the study) was 35% (29/83). Of those randomized to the Calm app, 55% (15/29) were men. Retention among men was higher (14/15, 93%) than that among women (12/20, 60%).

### Stress and Related Outcomes

No significant within or between group differences in stress or psychological outcomes related to stress were observed, nor were significant differences observed in health behaviors related to stress (Table 6).

**Table 6:** Pre- and postintervention values for outcome measures.

Variable		Calm group (n=33), mean (SD)	*t* test (*df*=32)		*P* value		POD group (n=27), mean (SD)		*t* test (*df*=26)		*P* value		*F* test (*df*=58)		*P* value	
**Stress (PSS^a^)**				1.8		.58				–0.2		.82		0.3		.86
	Preintervention	19.2 (7.3)					18.8 (8.0)									
	Postintervention	19.9 (8.1)					19.1 (6.4)									
**Anxiety (HADS^b^)**				–0.3		.77				–1.7		.10		2.2		.28
	Preintervention	8.8 (4.3)					7.2 (2.8)									
	Postintervention	8.9 (4.4)					8.2 (3.5)									
**Depression (HADS)**				–0.6		.58				–2.1		.04		7.1		.47
	Preintervention	5.8 (3.4)					4.5 (3.4)									
	Postintervention	6.5 (3.5)					5.7 (3.9)									
**Mindfulness (MAAS^c^)**				1.9		.07				1.0		.32		4.1		.55
	Preintervention	56.6 (14.0)					59.9 (10.3)									
	Postintervention	53.7 (13.6)					58.3 (10.3)									
**Physical Activity (IPAQ^d^)**				1.5		.13				–0.6		.56		1.1		.44
	Preintervention	706.9 (383.1)					753.9 (335.6)^e^									
	Postintervention	637.2 (315.6)					766.4 (396.4)^e^									
**Eating Habits (SSES^f^)**				–1.9		.07				0.1		.95		1.9		.15
	Preintervention	28.8 (9.6)					29.7 (8.5)									
	Postintervention	31.6 (10.7)					29.6 (8.8)									
**Coping (Brief COPE^g^)**				1.2		.23				1.5		.16		3.3		.98
	Preintervention	64.8 (9.2)					64.8 (9.3)^e^									
	Postintervention	62.8 (12.1)					62.7 (8.9)^e^									

^a^PSS: Perceived Stress Scale.

^b^HADS: Hospital Anxiety and Depression Scale.

^c^MAAS: Mindful Attention Awareness Scale.

^d^IPAQ: International Physical Activity Questionnaire.

^e^One POD participant did not complete the survey.

^f^SSES: Salzburg Stress Eating Scale.

^g^COPE: Coping Orientation to Problems Experienced (Scale).

### COVID-19 Survey

Most participants in Calm reported that the COVID-19 pandemic affected their stress levels (26/28, 93%), mental health (23/28, 82%), and physical health (17/28, 61%) (Table 7).

**Table 7:** COVID-19 survey results.

Question	Value, n (%)	
	Calm group (n=28)^a^	POD group (n=23)^b^
Pandemic has affected stress	26 (92.9)	22 (95.7)
Pandemic has affected mental health	23 (82.1)	20 (87)
Pandemic has affected physical health	17 (60.7)	17 (73.9)
Perception of personal risk to be high	5 (17.9)	3 (13)
Perception of personal risk to be higher than others in the United States	4 (14.3)	3 (13)
Ability to prevent contracting COVID-19 is high	11 (39.3)	11 (47.8)
Ability to prevent contracting COVID-19 is higher than others in the United States	9 (32.1)	12 (52.2)
Ability to prevent contracting COVID-19 is higher than other infectious diseases	5 (17.9)	7 (30.4)
Personally worried about contracting COVID-19	22 (78.6)	20 (86.9)
Worried about a family member contracting COVID-19	26 (92.9)	22 (95.7)
Worried about the spread of COVID-19	24 (85.7)	22 (95.7)

^a^Five participants did not complete the survey.

^b^Four participants did not complete the survey.

## Discussion

### Principal Findings

A 4-week, app-based mindfulness meditation intervention (ie, Calm app) may be feasible for use among middle-aged adults. Calm group participants expressed satisfaction with the intervention and felt it was appropriate and useful. However, significant improvements in perceived stress and psychological outcomes (ie, anxiety, depressive symptoms, mindfulness, and general coping) or health behaviors related to stress (ie, physical activity and eating habits) were not observed among these participants. The majority of participants reported that COVID-19 has negatively affected their stress, mental health, and physical health.

### Feasibility of Calm

We exceeded our benchmark (>75%) for acceptability rating of the Calm app among middle-aged adults experiencing stress. This finding was similar to that of other studies assessing the feasibility of Calm in patients with cancer and among college students [25]. Our benchmark for adherence to the meditation prescription (ie, >70% of the sample who completed at least 70% of the meditations) was met and better than most 4-week randomized controlled trials using an app to reduce stress [13,23,40]. Men had a higher adherence to the intervention than women (86% vs 71%), but this finding is not entirely consistent with other studies and does not necessarily suggest overall gender differences in meditation app use [41,42]. Research on app-based interventions targeting stress reduction and related outcomes, including objective app-usage data in middle-aged men, is warranted.

Our benchmark was to recruit 40% men, and we recruited 35% men (29/83) in our study. We were able to recruit more men than other app-based meditation studies (5.7%-27%), including studies that focused on middle-aged adults [21,25,43-46]. The retention of men (14/15, 93%) was also higher than that of women (19/24, 60%). Although, on average, men spent less time meditating than women did (approximately 57-91 vs 107-135 min/week) and completed fewer weekly sessions (4.7-7.5 vs 7.4-9.7 sessions/week), they were more likely to adhere to the 10-minute prescription. This finding suggests that Calm may be a useful self-management tool for both men and women to manage stress [21,44,45]. Future app-based meditation interventions should focus on recruitment and retention of men, especially because men are less likely to seek stress management strategies than women [47,48].

### Stress and Health-Related Behaviors

Significant changes in stress and related psychological outcomes or health behaviors were not observed. In another study testing the efficacy of meditation delivered via Calm, changes in stress and mindfulness in college students were observed after 8 weeks of participation. When taken together with these findings, the present data suggest that any significant changes in stress levels may take longer than 4 weeks to be noticeable [25].

Few studies have tested the effects of app-based mindfulness meditation on health behaviors related to stress [49] and have reported inconsistent findings [23,40,50,51]. We observed a negative trend regarding physical activity and eating habits. These may, however, have been related to lifestyle modifications due to the COVID-19 pandemic (eg, quarantine and closure).

### Perceptions of COVID-19

Perceptions of COVID-19 could have had an important impact on stress in relation to the findings of this study. It is possible that meditation helped the study participants in the intervention group to maintain their stress and anxiety levels, as well as depressive symptoms (as opposed to elevated levels), during the COVID-19 pandemic [52]. Likewise, the general health education content of the control app may have helped mitigate the impact of the pandemic through avoidance or redirecting negative thoughts [53]. However, data regarding how participants felt their app usage impacted their COVID-19–related stress was not measured. More research on the effects of COVID-19 on stress and related health outcomes and how a meditation app may buffer that impact is warranted.

### Limitations

Limitations to this study include the following: (1) the majority of the sample comprised White participants, and generalizability of the findings may thus be limited; (2) the intervention was only 4 weeks long and did not include a follow-up; and (3) the COVID-19 pandemic may have had a notable impact on the findings of this study.

### Conclusions

This study supports the feasibility of a 4-week, mobile app–based mindfulness meditation intervention (ie, Calm) in middle-aged men and women with specific application for the recruitment of men to inform future studies.

## Appendix

Multimedia Appendix 1 CONSORT-eHEALTH checklist (V 1.6.1).

## Abbreviations

RedCap: Research Electronic Data Capture
